# Antimicrobial Evaluation of Two Polycyclic Polyprenylated Acylphloroglucinol Compounds: PPAP23 and PPAP53

**DOI:** 10.3390/ijms25158023

**Published:** 2024-07-23

**Authors:** Aparna Viswanathan Ammanath, Miki Matsuo, Huanhuan Wang, Frank Kraus, Anton Bleisch, Philipp Peslalz, Majd Mohammad, Meghshree Deshmukh, Anne Grießhammer, Moushumi Purkayastha, Andreas Vorbach, Boris Macek, Heike Brötz-Oesterhelt, Lisa Maier, Dorothee Kretschmer, Andreas Peschel, Tao Jin, Bernd Plietker, Friedrich Götz

**Affiliations:** 1 Microbial Genetics, Interfaculty Institute of Microbiology and Infection Medicine Tübingen (IMIT), University of Tübingen, 72076 Tübingen, Germany; 2 Organic Chemistry I, Faculty of Chemistry and Food Chemistry, Technical University Dresden, 01062 Dresden, Germanyphilipp.peslalz@tu-dresden.de (P.P.);; 3 Department of Rheumatology and Inflammation Research, Institute of Medicine, Sahlgrenska Academy, University of Gothenburg, 40530 Gothenburg, Sweden; majd.mohammad@rheuma.gu.se (M.M.); meghshree.vinod.deshmukh@gu.se (M.D.);; 4 Interfakultäres Institut für Mikrobiologie und Infektionsmedizin (IMIT), University of Tübingen, 72076 Tübingen, Germany; 5 Excellence Cluster 2124 ‘Controlling Microbes to Fight Infections’ (CMFI), University of Tübingen, 72076 Tübingen, Germany; 6 Microbial Bioactive Compounds, Interfaculty Institute of Microbiology and Infection Medicine Tübingen (IMIT), University of Tübingen, 72076 Tübingen, Germany; andreas.vorbach@med.uni-tuebingen.de; 7 Quantitative Proteomics, Proteome Center Tübingen, Interfaculty Institute for Cell Biology, University of Tübingen, 72076 Tübingen, Germany

**Keywords:** albumin, antimicrobial activity, larvae, mouse septic arthroses, PPAP, gastrointestinal anaerobes

## Abstract

Polycyclic polyprenylated acylphloroglucinols (PPAPs) comprise a large group of compounds of mostly plant origin. The best-known compound is hyperforin from St. John’s wort with its antidepressant, antitumor and antimicrobial properties. The chemical synthesis of PPAP variants allows the generation of compounds with improved activity and compatibility. Here, we studied the antimicrobial activity of two synthetic PPAP-derivatives, the water-insoluble PPAP23 and the water-soluble sodium salt PPAP53. In vitro, both compounds exhibited good activity against methicillin-resistant *Staphylococcus aureus* (MRSA) and vancomycin-resistant *Enterococcus faecium*. Both compounds had no adverse effects on *Galleria mellonella* wax moth larvae. However, they were unable to protect the larvae from infection with *S. aureus* because components of the larval coelom neutralized the antimicrobial activity; a similar effect was also seen with serum albumin. In silico docking studies with PPAP53 revealed that it binds to the F1 pocket of human serum albumin with a binding energy of −7.5 kcal/mol. In an infection model of septic arthritis, PPAP23 decreased the formation of abscesses and *S. aureus* load in kidneys; in a mouse skin abscess model, topical treatment with PPAP53 reduced *S. aureus* counts. Both PPAPs were active against anaerobic Gram-positive gut bacteria such as neurotransmitter-producing *Clostridium*, *Enterococcus* or *Ruminococcus* species. Based on these results, we foresee possible applications in the decolonization of pathogens.

## 1. Introduction

Hyperforin is one of the main ingredients of and most likely the one responsible for the antidepressant, antitumoral and antimicrobial effects of St. John’s wort [1,2,3]. Hyperforin belongs to the family of polycyclic polyprenylated acylphloroglucinols (PPAPs) and is a rather unstable compound [4]. However, a hydrogenated hyperforin analogue showed increased stability and antimicrobial activity against various Gram-positive bacteria [5,6]. A breakthrough in PPAP research was achieved with the total synthesis of >100 defined endo-type B PPAP compounds and of non-natural PPAP analogs [7,8,9,10]. Very recently, 30 additional entities based on the structure–activity relationship against methicillin-resistant *Staphylococcus aureus* (MRSA) were synthesized [11]. All of these compounds were evaluated for their antibacterial activity and cytotoxicity.

Four of the new compounds, namely, PPAP 14, 19, 22, and 23, showed high activity against multiple resistant Gram+ pathogens such as MRSA and vancomycin-resistant *Enterococcus faecium* and comparatively low toxicity against HL60 cells [12,13]. Despite repeated attempts, no PPAP23-resistant mutants were isolated, which is advantageous for a potential application.

The mode of action of PPAP23 was primarily investigated in MRSA [13]. Transcriptional analyses indicate that PPAP23 signals excess iron to the bacterial cells, as genes involved in iron transport are downregulated, and genes involved in iron storage are upregulated. The excess iron reaction can be explained by the mild iron chelating activity of PPAP23. The antimicrobial activity of PPAP23 mainly manifests in low-iron medium and is attenuated by the addition of exogenous iron. It is of particular interest that while impairing membrane integrity, PPAP23 does not lead to pore formation. Rather, PPAP23 preferentially inhibits Fe-S cluster enzymes in the membrane and, thus, also respiration. All data indicate that PPAP23 with its hydrophobic pocket interacts with the cytoplasmic membrane and interferes with iron metabolism.

Besides PPAP23, recently, PPAP 22 was described which exhibits very good activity against multiple resistant *S. aureus* strains and low IC50 values [12]. Based on PPAP 22, the sodium salt derivative PPAP53 was produced, which showed significantly improved water solubility [14]. PPAP53 was tested against multi-drug resistant *Mycobacterium tuberculosis* (Mtb) and was found to inhibit the growth of virulent extracellular and intracellular Mtb without damaging primary human macrophages [14]. PPAP53 could be the founding member of a new class of antimycobacterial compounds.

However, previous studies on the activity of PPAP23 and PPAP53 have largely been conducted in vitro or in cell cultures and are therefore of limited value for potential therapeutic application. The aim of this work was therefore to investigate the efficacy of these two compounds in different in vivo models.

Here, we show that PPAP23 and the more water-soluble sodium salt PPAP53 have good antimicrobial properties against Gram+ pathogens, and both PPAP23 and 53 are very well tolerated in the insect and mouse models. However, they are unable to effectively protect against *S. aureus* infection. The main reason for this is that the antimicrobial activity of both PPAPs is neutralized by components of the larval coelom and by mammalian albumin. We see a possible therapeutic application in an albumin-deficient milieu, for example, in the context of certain gastrointestinal (GI) infections, as PPAP23 has a selective efficacy against intestinal, anaerobic Gram-positive bacterial pathogens.

## 2. Results

### 2.1. The PPAP Compounds 23 and 53

**Figure 1:**
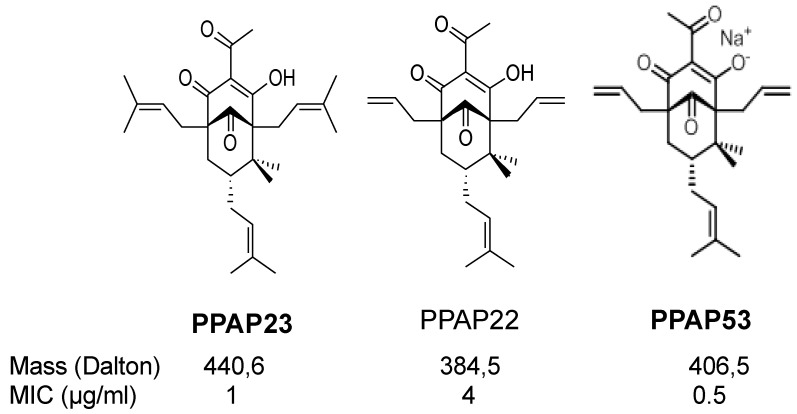
Structures of PPAP23, PPAP 22 and PPAP53. PPAPs exhibit similar MIC values (1 to 2 μg/mL) against the multi-resistant *S. aureus* USA300 strain. PPAP53, being the sodium salt form of PPAP 22, is more soluble in water and was dissolved accordingly. On the other hand, PPAP23 was dissolved using DMSO.

Similar to hyperforin, many of the synthesized derivatives are only poorly soluble in water. For example, the well-studied PPAP 22 and 23 were soluble in 100% DMSO, 100% ethanol, 80% methanol, 8.2% cyclodextrin/18% DMSO in PBS and 0.4% Tween 80/18% DMSO in PBS. Since high concentrations of DMSO, methanol, ethanol and β-cyclodextrin are cytotoxic, we selected 0.4% Tween 80/18% DMSO in PBS as a suitable solvent. PPAP23 was used at a stock concentration of 10 mg/mL dissolved in 50% DMSO. For our studies, we ensured that the final working concentrations of DMSO were significantly below 1%. As shown in Figure 1, PPAP 22 and PPAP23 chemically differ in the R2 position: PPAP23 has a prenyl residue, while in PPAP 22, the R2 position is occupied by an allyl residue [12]. When tested towards *S. aureus* USA300, the MIC values of PPAP 22 and PPAP23 were 2 µg/mL and 1 µg/mL, respectively. When tested towards the human monocytic cell line HL-60, the IC_50_ values of PPAP 22 and PPAP23 were 223 µg/mL and 96 µg/mL, respectively [13]. Hence, despite a lower MIC, PPAP23 exhibits higher cytotoxicity than PPAP22. For the investigation of the mode of action, we have previously focused on PPAP23 [13]. To improve the solubility of PPAPs, a sodium salt derivative of PPAP 22 was generated, namely, PPAP53 (Figure 1). 1H- and 13C-NMR spectra of PPAP53 and HPLC purity are shown in Figures S1 and S2. In this study, we mainly aimed at investigating whether the water-soluble PPAP53 displayed higher in vivo activity than the less water-soluble PPAP23.

### 2.2. PPAP23 and PPAP53 Have No Toxic Effects on Larvae but Could Not Rescue Larvae in an Infection Model

**Figure 2:**
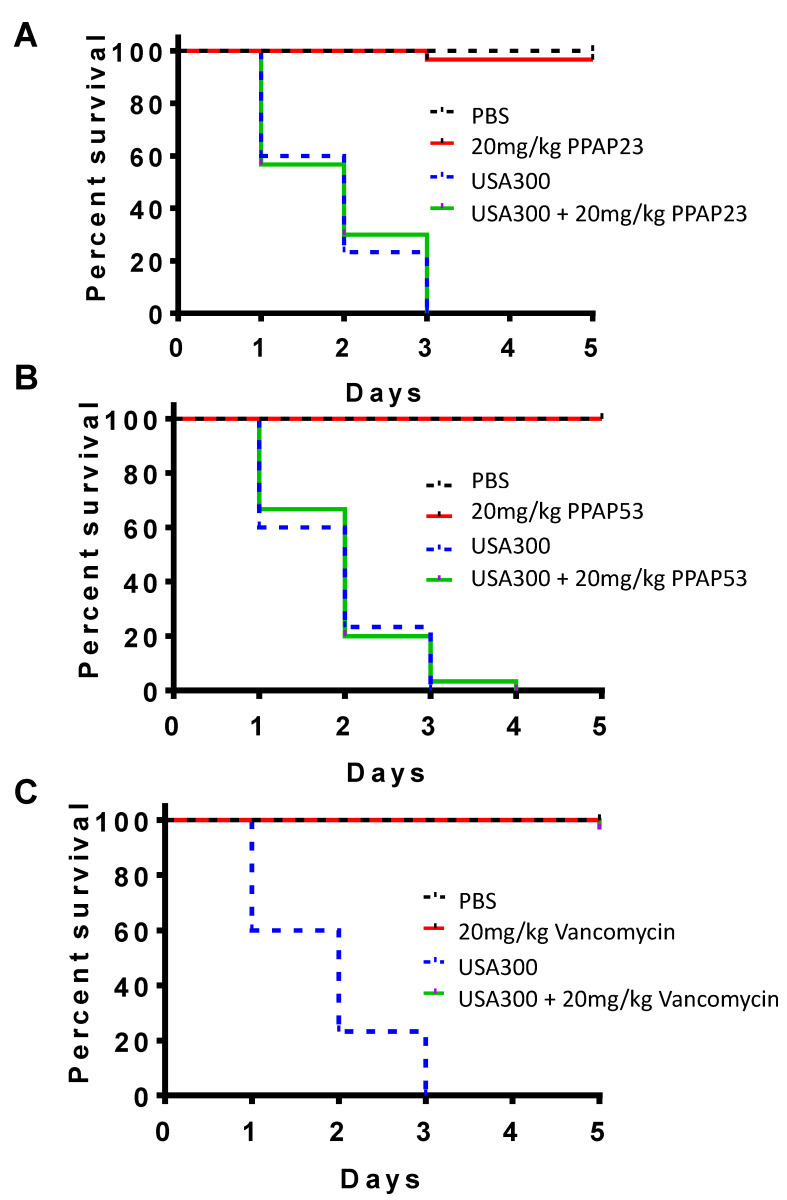
PPAP23 and PPAP53 were found to be non-toxic to larvae, but they did not protect the larvae from infection with *S. aureus* USA300. Groups of ten *Galleria mellonella* larvae, each weighing about 500 mg, were used for the experiment. The groups included untreated larvae, larvae injected with 10^6^ cfu of *S. aureus* USA300 (in the last right proleg) and larvae treated one hour after bacterial administration with either 20 mg/kg (45 μM) PPAP23 (**A**), 20 mg/kg (50 μM) PPAP53 (**B**), or vancomycin (**C**) at a similar dose (20 mg/kg, 13 μM). In the absence of treatment, the infected larvae typically succumbed to *S. aureus* within three days. The larvae were kept at 37 °C and monitored daily for mortality over five days. The results shown in the graph represent three biological replicates.

The antibacterial activity of PPAP23 and PPAP53 was tested in *Galleria mellonella* larvae infected with *S. aureus* USA300, using a dosage of 20 mg/kg (20 × MIC) for both compounds. When the PPAPs were injected alone, all the larvae survived, showing that PPAP23 and 53 are well tolerated at this dosage (Figure 2A,B). However, when the larvae were infected with 10^6^ CFU *S. aureus* USA300, all larvae died within 3 days. Treatment with either PPAP23 or PPAP53 (dosage: 20 mg/kg (50 μM)) one hour after bacterial infection did not increase the viability of the infected larvae (Figure 2A,B). As a control, we used a comparable dose of vancomycin (20 mg/kg, 13 μM), which completely protected the USA300-infected larvae (Figure 2C). Since PPAP23 and PPAP53 exhibited good antimicrobial activity in vitro, we asked ourselves why in the in vivo studies PPAPs were not effective.

### 2.3. The Coelomic Fluid of the Larvae Antagonizes the Activity of PPAP23 and PPAP53

**Figure 3:**
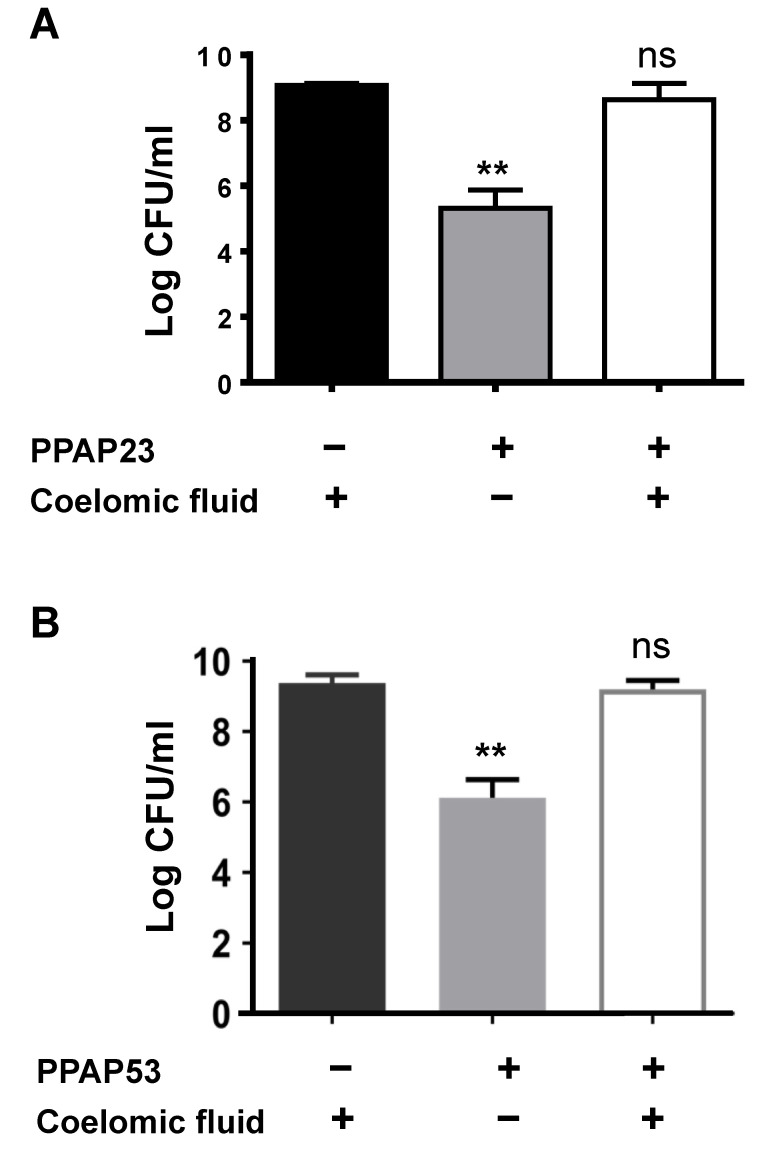
The bactericidal activity of PPAP23 and PPAP53 is neutralized by larval coelomic fluid. To simulate the in vivo larval infection scenario, an ex vivo killing assay was conducted. A bacterial inoculum of 10^5^ CFU and 10 μg of either (**A**) PPAP23 or (**B**) PPAP53 was added to 100 μL of larval coelomic fluid for the treatment group. Controls included untreated larval fluid and larval fluid treated with PBS. Bacterial viability in each group was assessed using the drop plate method. The coelomic fluid reversed the bactericidal effect of both PPAP23 and PPAP53 on *S. aureus*. ns indicates a non-significant result (*p* > 0.05), and ** denote *p* < 0.01.

We suspected that coelomic fluid neutralized the effect of PPAPs. So, to mimic the in vivo larval experiment, 10 µg of PPAP23 and PPAP53 was added to 100 μL of sterile larval coelomic fluid and then incubated with 10^6^ CFU/mL S. aureus USA300 overnight. PPAP23 or PPAP53 and USA300 incubated with PBS served as positive controls. As suspected, no antibacterial activity of PPAP23 and PPAP53 towards S. aureus was observed in the presence of the coelomic fluid (Figure 3A,B). This suggests that components in the larval coelomic fluid antagonize the activity of PPAPs. This raised the question whether mammalian serum and albumin, one of the main proteins of blood plasma, could also neutralize the antibacterial activity of PPAPs.

### 2.4. Bovine Serum and Albumin Abrogate the Bactericidal Activity of PPAP53

**Figure 4:**
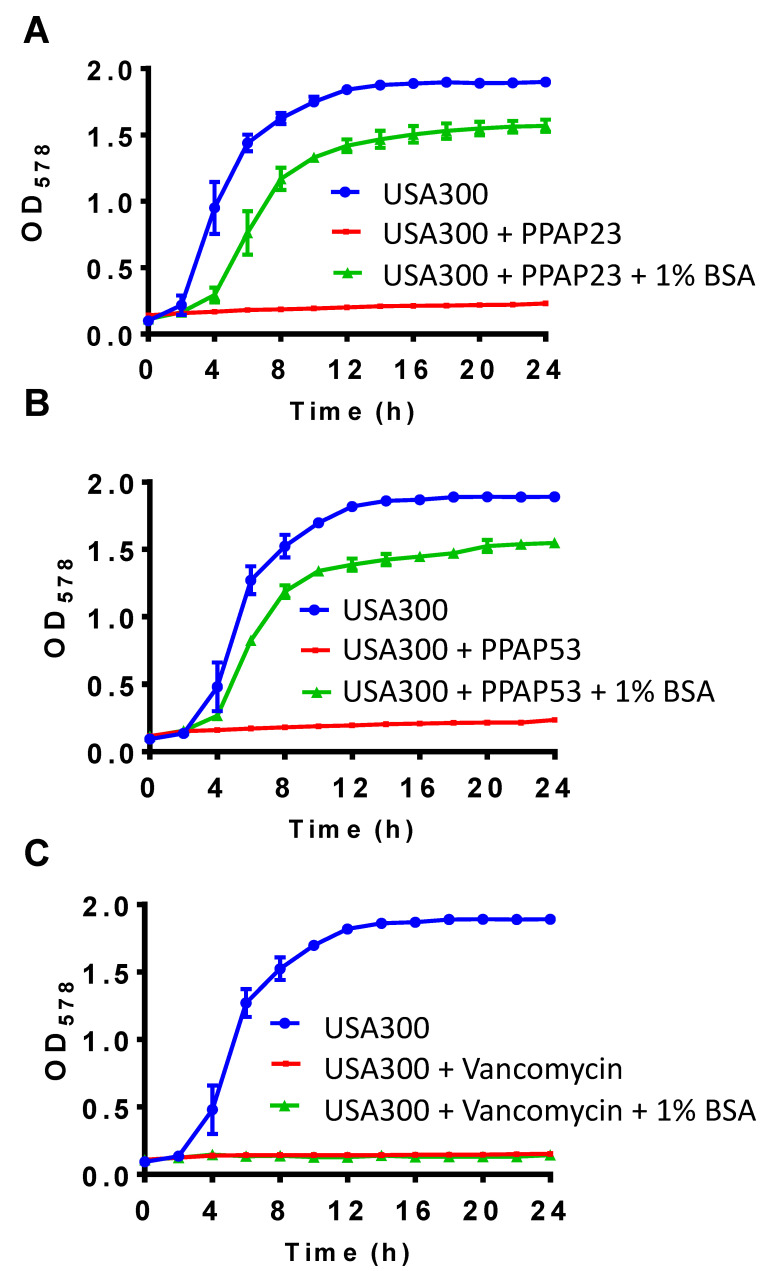
The bactericidal activity of PPAP23 and PPAP53 is reversed by 1% bovine serum albumin (BSA). *S. aureus* cells grown overnight in TSB were adjusted to OD = 0.01 in a 48-well plate, and 1X MIC PPAPs and vancomycin with or without 1% BSA were added to the culture. The bacterial growth was measured every 2 h using Varioskan LUX Multimode Microplate Reader. The bactericidal effect of (**A**) PPAP23 and (**B**) PPAP53 (MIC: 0.5–1 µg/mL) on *S. aureus* USA300 was reversed by 1% BSA, whereas the effect of (**C**) vancomycin remained unchanged.

The larval coelomic fluid is very similar to the mammalian blood serum [15]. As coelomic fluid neutralizes the antimicrobial activity of PPAP53, we were curious to determine whether the mammalian serum and its components (mainly serum albumin, immunoglobulin (IgG) and fibrinogen (Fg)) also showed a similar effect. For this, we determined the MIC of PPAP53 on USA300 in the presence of fetal bovine serum (FBS). We could see a 32-fold reduction in the antibacterial activity of PPAP53 in the presence of 25% FBS (Table 1). Bovine serum albumin (BSA) (10 mg/mL) also significantly decreased the in vitro antimicrobial activity of PPAP53, while IgG and Fg (at concentrations up to 25 mg/mL) had no effect (Table 1). Hence, albumin is the main protein in FBS that abrogates the bactericidal activity of PPAP53. The growth kinetic studies of USA300 in the presence of PPAPs and/or 1% BSA revealed that the antimicrobial effect of the PPAPs was neutralized by 1% BSA (Figure 4A,B). The antibacterial activity of vancomycin remained unaffected in the presence of 1% BSA (Figure 4C).

### 2.5. In Silico Docking Studies Show PPAP53 Binds to the Hemin Binding Pocket of HSA

Serum albumin, the most prevalent plasma protein (~640 μM), can bind various hydrophobic ligands such as fatty acids, bilirubin, thyroxine and hemin [16]. To investigate the binding of PPAP53 to serum albumin, we conducted molecular dynamics simulations. We downloaded a well-defined crystal structure of human serum albumin (HSA) from the Protein Data Bank (PDB ID: 1E78), removing the calcium and acetate ions and crystallization water molecules to obtain a clean HSA structure.

Using Chem3D 16.0, we prepared a 3D structure of PPAP53 from its 2D structure and optimized its geometry through energy minimization. The structures were visualized and verified with PyMOL 3.0 software. We prepared PDBQT files for HSA and the ligands using AutoDock Tools, adding polar hydrogens and Kollman charges. Blind docking was then performed using AutoDock Vina (https://vina.scripps.edu/, accessed on 23 July 2013), treating HSA as a rigid structure and PPAP53 as flexible.

**Figure 5:**
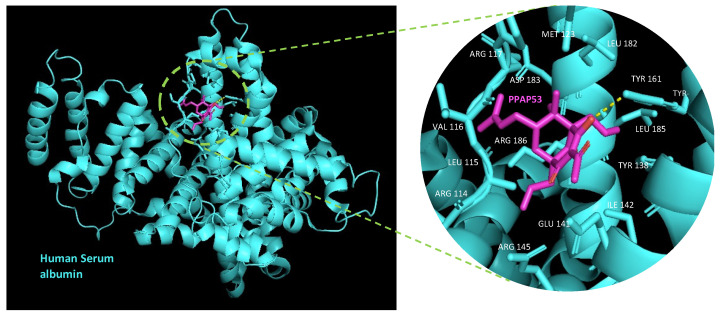
PPAP53 binds to the hemin binding pocket of HSA. In silico docking analysis by AutoDock vina showed that PPAP53 binds to FA1 pocket of HSA with a binding energy of −7.5 kcal/mol. This pocket represents the third main ligand (e.g., drug) binding pocket of HSA, hemin being a prototypical ligand. HSA is displayed in a cartoon style whereas PPAP53 and the HSA residues lining the pocket are presented as capped sticks. Amino acids surrounding the pocket include ARG114, LEU115, VAL116, ARG117, PRO118, MET123, TYR138, GLU141, ILE142, ARG145, TYR161, LEU179, LEU182, ASP183, LEU185, ARG186 and GLY189.

The in silico docking analysis revealed that PPAP53 binds to the IB pocket (His128, His146 = subdomain IB) of HSA with a binding energy of −7.5 kcal/mol (Figure 5). This IB pocket is the third main ligand binding site of HSA, typically binding ligands like hemin, azapropazone, indomethacin and 3,5-triiodobenzoic acid [17]. PPAP53 shows a hydrogen bond interaction with TRY161 of HSA.

### 2.6. Addition of Known Albumin Ligands Does Not Improve the In Vitro Antibacterial Activity of PPAP53

Since PPAP53 binds a well-characterized ligand binding pocket of HSA, we reasoned that known ligands could compete with PPAP53 for binding to HSA, thus relieving inhibitory effects of HSA on PPAP53 antimicrobial activity. However, the co-incubation of PPAP53 with FDA-approved albumin drugs did not restore the antibacterial activity of PPAP53. The binding sites of different FDA drugs (ligands) [17] on albumin are given in Table S1. The effects of PPAP53 in combination with different albumin ligands in 1% BSA on the growth kinetics of USA300 are shown in Figures S3–S5.

### 2.7. PPAP53 Reduces Growth of S. aureus USA300 in Subcutaneous Abscesses

**Figure 6:**
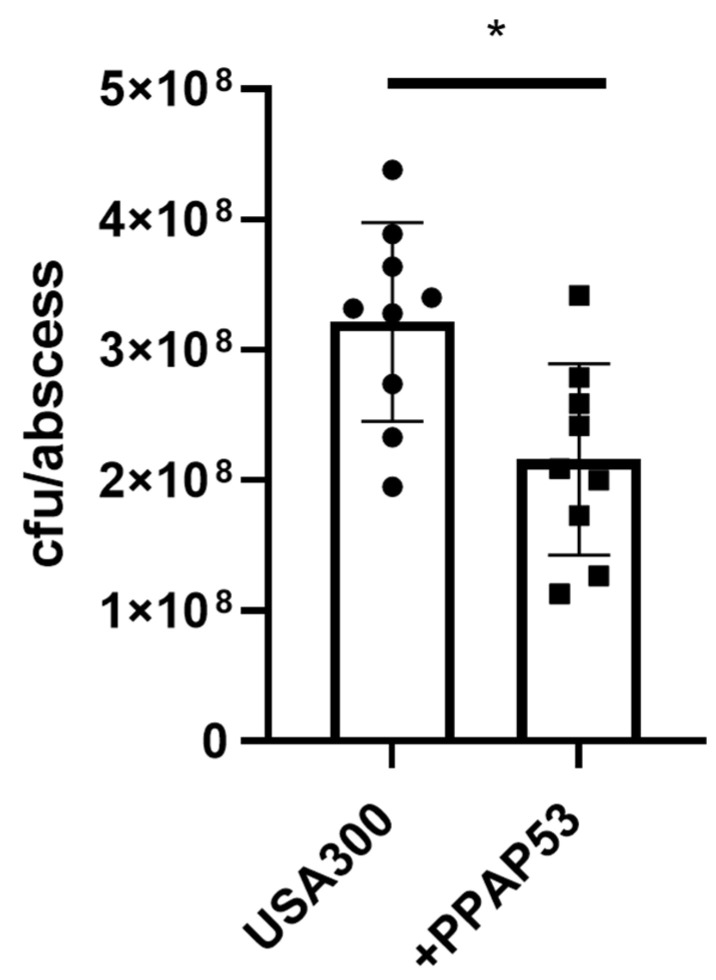
PPAP53 treatment significantly reduced bacterial load in skin abscesses. C57BL6J mice s.c. inoculated with *S. aureus* USA300 strain (105 colony-forming units/mouse) and sterile dextran beads were treated twice, two hours and 24 h after infection with PPAP53 dissolved in PBS (500 µg/kg) or with PBS (*n* = 9). After 48 h, the abscesses were excised and homogenized, and CFUs were determined. Statistical evaluations were performed using the Mann–Whitney U test. Data are mean values ± standard error of the mean. * *p* < 0.05.

Since serum albumin inhibits the activity of PPAP53, we analyzed whether topical application of PPAP53 could inhibit bacterial growth in skin abscesses. To this end, we used a mouse skin abscess model that closely resembles staphylococcal skin infections in humans [18]. Since USA300 is known to induce severe skin infections, we induced skin abscesses by subcutaneous injection of USA300 into the flanks of mice. Interestingly, the topical application of PPAP53 on filter paper disks two hours and 24 h after infection significantly reduced bacterial loads in skin abscesses (Figure 6 and Figure S2), indicating that PPAP53 can penetrate skin tissues and reach subcutaneous abscesses to limit bacterial growth.

### 2.8. PPAP23 Shows a Beneficial but Not Fully Protective Effect on S. aureus Septic Arthritis Mouse Model

**Figure 7:**
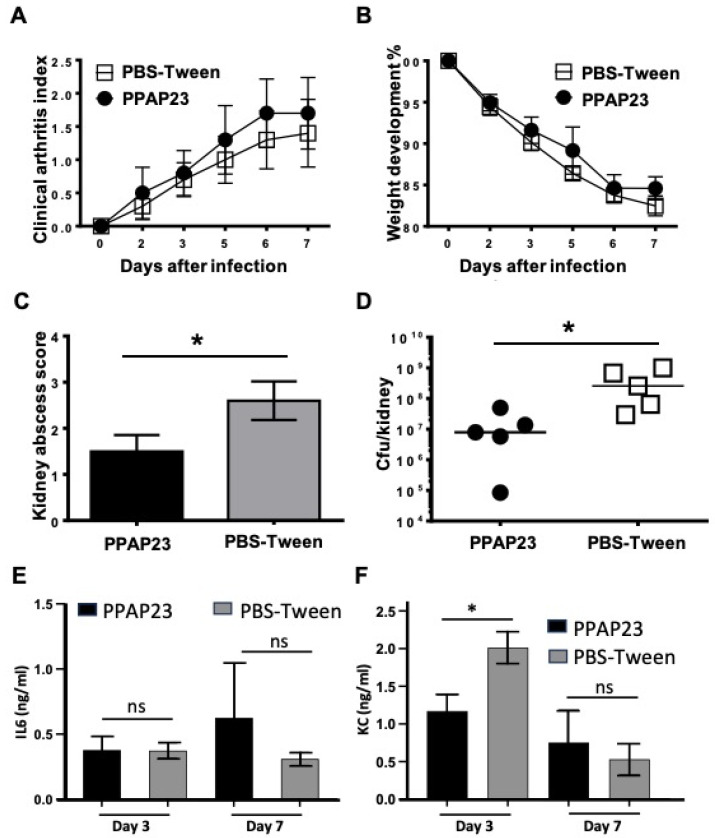
Treatment with PPAP23 markedly decreased abscess formation and bacterial load in the kidneys of mice suffering from *S. aureus*-induced septic arthritis. NMRI mice, injected with the *S. aureus* Newman strain (4 × 10^6^ CFU per mouse), received PPAP23 in 0.5% Tween 80 in PBS (100 µg per mouse; n = 5) or the same volume of 0.5% Tween 80 in PBS (n = 5) twice daily, starting on day 2 post-inoculation until euthanasia on day 7. The severity of arthritis symptoms (**A**) and changes in body weight (**B**) were monitored for 7 days after infection. Kidney abscess scores (**C**) and bacterial presence in kidneys (**D**) were assessed in mice euthanized 7 days after infection. Blood levels of IL-6 (**E**) and KC (**F**) were measured on days 3 and 7 post-infection. Statistical analysis was conducted using the Mann–Whitney U test, with data presented as mean values ± standard error of the mean. * *p* < 0.05; ns = not significant.

Since we obtained different results in the insect and mammalian model for PPAP53, we checked whether PPAP23 had a protective effect in the mouse model of septic arthritis caused by *S. aureus* Newman. PPAP23 was injected intraperitoneally at a dose of 100 μg per mouse twice daily from day 2 of infection with *S. aureus* Newman. Visible symptoms of septic arthritis started on day 2 after infection and worsened up to day 7. We did not observe any difference between the clinical severity of septic arthritis in mice treated with PPAP23 and the control group (mice treated with vehicle (0.5% Tween 80 in PBS) (Figure 7A). During the span of the study, we also did not observe any significant difference in the loss of body weight of mice in the two groups (Figure 7B). Nevertheless, the abscess core in the kidneys of mice treated with PPAP23 and the cfu/kidney were significantly lower than those of mice treated with the vehicle (*p* < 0.05; Figure 7C). The bacterial load in the kidneys of PPAP23 treated mice was >25-fold lower than the one of mice treated with the vehicle (*p* < 0.05; Figure 7D). The bacterial load and the abscess score in the kidneys were significantly correlated (r = 0.95; *p* < 0.001). To study the proinflammatory immune response, we analyzed both the levels of IL6 and keratinocyte-derived chemokine (KC), a murine IL8 homologue, in blood from both groups of mice. No significant differences between groups were observed for IL6 levels (Figure 7E) in either the early or later stages of infection. However, significantly lower levels of KC in PPAP23-treated mice were observed in the early stage of infection as compared with controls (*p* < 0.05; Figure 7F), and this difference disappeared by day 7 post-infection. This indicates that PPAP23 treatment had a slight, but favorable effect on the clearance of *S. aureus* in septic arthritic mice.

### 2.9. PPAP23 and PPAP53 Show Activity against Gut, Gram+ Anaerobic Pathogens

We previously showed that PPAP23 and 53 has good antibacterial activity against aerobic and facultative aerobic Gram+ bacteria [13]. The effect of PPAPs on anaerobic gut microbiota has not been studied so far. Here, we found that PPAP23 and 53 had good bactericidal activity against various Gram+ anaerobic pathogenic gut bacteria such as *Clostridium perfringens*, *Clostridium difficile* and other disease-associated species (Table 2), while some commensal anaerobic gut species were unaffected by PPAP23 and 53.

## 3. Discussion

The polycyclic polyprenylated acylphloroglucinols (PPAPs) represent a large group of natural products. In particular, the diverse biological activity of these compounds has spurred chemical synthesis and concomitant derivatization. The current database comprises more than 850 structures with different bioactivities [3,19,20,21,22]. The development of a seven-step synthetic approach enabled the total synthesis of a large variety of structurally diverse endo-type B PPAPs [7,8]. In previous studies, we mainly focused on the antimicrobial mode of action of PPAP23. We were able to show that PPAP23 mainly targets the bacterial cytoplasmic membrane of Gram+ bacteria, inactivates Fe-S cluster enzymes and induces a breakdown of the bacterial membrane potential [12,13]. Because of their small size (~400 Da), PPAP23 and 53 represent good drug candidates.

Here, we focused more on the in vivo activity of PPAP23 and included in our study the new water-soluble sodium salt derivative of PPAP 22, named PPAP53 (Structures in Figure 1). As expected, the MIC (µg/mL) of PPAP53 against *S. aureus* USA300 was lower than PPAP23 (0.5 compared to 1.0; Table 1). The first animal model in which we tested the two PPAPs was the wax moth larval model of *Galleria mellonella*, which is widely used to study the infectivity of pathogenic bacteria or the in vivo efficacy of antibiotics [23]. Both PPAPs were well tolerated by larvae at the concentration used, suggesting that the PPAPs do not induce toxic side effects. The synthetic PPAPs 23 and 53 are not only structurally related to hyperforin but also share other properties. Like hyperforin, PPAP23 is highly hydrophobic, poorly water soluble and binds with high affinity to serum albumin, which affects its anti-inflammatory effect [24,25]. The advantage of PPAP23 over hyperforin is that it has lower cytotoxicity and is photochemically stable; PPAP23 displayed an IC50 value of only 96 µg/mL, which is 15-fold less toxic than hyperforin [26].

However, when the larvae were challenged with USA300, both PPAPs failed to protect the infected larvae from being killed by USA300 because of their neutralization by components of the coelomic fluid. Unfortunately, BSA also neutralized PPAPs, while other common serum proteins such as fibrinogen or immunoglobulins showed no neutralizing effect (Table 1). Despite this not-so-promising perspective, and because the insect system and the mammalian system are significantly different, we investigated the efficacy of PPAPs in two different mouse models. In the mouse skin abscess model, *S. aureus* causes a typical skin disease which allows the testing of antimicrobial agents by targeted application to mouse skin. First, we showed that topical treatment of the skin with PPAP53 did not cause local or systemic signs of toxicity, indicating that PPAP53 was skin-tolerable. To generate skin abscess, USA300 was injected subcutaneously into the shaved flanks of mice after adsorption to silica nanospheres to hold the bacteria in a specific location in the skin tissue and allow abscess formation. The topical treatment with PPAP53 caused a twofold reduction in bacterial counts (Figure 5). Since the bacteria were administered subcutaneously but PPAP53 was applied on the surface of the skin, it can be assumed that PPAP53 can also penetrate and show activity into deeper layers of the skin.

In the septic arthritis mouse model, we investigated the antibacterial activity of PPAP23. This compound did not lead to a full protection from USA300 infection; however, the formation of kidney abscesses was decreased, and also, the bacterial load was reduced. No difference was observed in IL6 levels on days 3 and 7 post-infection, in both PPAP23 treated and PBS-Tween treated mice. However, a significant difference was observed in KC levels in the early stage of infection, day 3, as compared to the later stage, day 7 post-infection. This indicates that an early immune response to infection, with neutrophil recruitment, is triggered in this mouse model, but not systemic inflammation.

Despite these positive effects of PPAP23 and PPAP53 in the two animal models, one must assume that neutralization by serum albumin undermines the efficacy of the two PPAPs in systemic application. With a concentration of 35–50 mg/mL in the human blood, serum albumin is the most abundant protein in vertebrates. Albumin is well known to bind to a broad spectrum of small molecules or compounds mainly via two binding sites in the subdomain IIA (site 1) and subdomain IIIA (site 2) [27,28]. Because of their small size and/or their hydrophobicity, PPAPs belong precisely to the class of compounds that are bound by albumin. Interestingly, the water-soluble Na salt of PPAP53 is neutralized by serum albumin almost as well as PPAP23, so we assume that the water-soluble Na salt does not offer any significant advantage in this respect.

While we have relatively high albumin levels in most body regions and organs, in the gastrointestinal tract, the albumin level is comparatively low, with only 0.01–0.24 mg/g wet weight of feces, which is 100 to 1000 times lower than the blood levels [29]. Furthermore, it has been demonstrated that when added to feces, albumin is degraded within a few hours [26]; the albumin concentration in the gastrointestinal tract of patients with inflammatory bowel disease (IBD) is even lower than in healthy individuals [13,30]. Therefore, it is likely that the antimicrobial activity of PPAPs is not impaired at such a low albumin concentration in the gastrointestinal tract.

With this in mind, we asked to what extent the PPAP23/53 inhibit the growth of anaerobic intestinal bacteria. Intriguingly, some of the most pathogenic anaerobic gastrointestinal (GI) bacteria were susceptible to PPAP23/53. These include *Clostridioides difficile* causing colitis and *Clostridium perfringens* causing diarrhea and food poisoning [31,32]. Other GI bacteria sensitive to PPAP23 were *Parabacteroides distasonis*, *Clostridium ramosum* and *Ruminococcus gnavus*. *Clostridium ramosum* is sometimes observed to cause bacteremia [33]. Reports show emerging antimicrobial resistance in *Parabacteroides distasonis*, an aerotolerant gut anaerobe with pathogenic and probiotic effects on human health [34]. *Ruminococcus gnavus* is associated with inflammatory bowel disease, particularly Crohn’s disease, and the production of inflammatory polysaccharides [35] (Table 2). Our results suggest the potential oral application of PPAPs for the treatment of intestinal dysbiosis caused by certain bacteria.

The inhibition of Gram-positive, anaerobic pathogenic intestinal bacteria by PPAP23 and PPAP53 is a desirable effect. But what is almost more interesting is that several representatives of this intestinal microbiota, such as strains of the *Clostridiaceae*, *Enterococcaceae*, *Lactobacillaceae* or *Ruminococcaceae* family, produce neurotransmitters, particularly trace amines [36,37]. This correlates with metagenomic analysis revealing that many commensal bacteria on the skin and gut have aromatic amino acid decarboxylases (AADCs), producing neurotransmitters which can interact with host neuroreceptors [38,39]. There is ample evidence that the skin and gut neurotransmitters communicate in multiple ways with the central nervous system (CNS) in a feedback process referred to as the “microbiota-brain-axis” [40,41,42,43]. PPAP23 and 53 may have a potential application in decreasing intestinal neurotransmitter production.

## 4. Materials and Methods

### 4.1. Bacterial Strains, Growth Conditions and Antibiotics

*S. aureus* USA300 was grown in Tryptic Soy Broth (TSB, Gibco, Bleiswijk, The Netherlands) at 37 °C with continuous shaking. MIC studies were carried out in Mueller Hinton Broth (MHB). *S. aureus* Newman AH5016 strain, a reporter strain [44], was received from Anschutz Medical Campus, University of Colorado, USA. The strain was cultivated for 24 h on horse blood agar plates and then stored as previously described [45]. Before each experiment, the bacterial solutions were thawed, washed and adjusted to the desired concentration for the respective experiments. Bovine serum albumin (BSA), fetal bovine serum (FBS), IgG from bovine serum and fibronectin from bovine plasma were purchased from Sigma, Darmstadt, Germany. For the MIC studies, FBS and BSA were dissolved in MHB to attain the desired concentrations, followed by sterile filtration. Bovine IgG and fibronectin were dissolved in 0.9% NaCl and sterile filtered before further dilution in MHB. PPAP 22, PPAP23 and PPAP53 were synthesized in the research group of Bernd Plietker. For BSA-interaction studies, we used FDA-approved ligands known to bind human serum albumin.

### 4.2. Synthesis of PPAP53

PPAP22 and PPAP23 were synthesized and spectroscopically characterized as previously described [11,12,14,46].

PPAP22 R_f_ = 0.63 (iso-hexane/ethyl acetate 9:1). ^1^H-NMR (600 MHz, CDCl_3_, 1.6:1 mixture of enol tautomers determined by ^1^H-NMR analysis) δ = 18.68 (s, 0.6H), 18.15 (s, 0.4H), 5.94–5.80 (m, 1H), 5.57–5.41 (m, 1H), 5.18–5.02 (m, 3H), 4.98–4.91 (m, 1H), 4.90–4.82 (m, 1H), 2.80 (dd, J = 13.2, 5.6 Hz, 0.6H), 2.68–2.48 (m, 3.4H), 2.61 (s, 1.8H), 2.58 (s, 1.2H), 2.24–2.02 (m, 3H), 2.00–1.79 (m, 1H), 1.65 (s, 3H), 1.48 (s, 1.8H), 1.45 (s, 1.2H), 1.48–1.38 (m, 1H), 1.20 (s, 1.8H), 1.18 (s, 1.2H), 1.02 (s, 1.8H), 0.98 (s, 1.2H) ppm. ^13^C-NMR (151 MHz, CDCl_3_, 1.6:1 mixture of enol tautomers determined by ^1^H-NMR analysis) δ = 207.6, 207.3, 200.9, 199.8, 199.5, 199.1, 194.5, 193.6, 134.2, 134.0, 133.6, 133.3, 133.1, 133.0, 123.7, 123.4, 119.3, 119.0, 118.8, 118.5, 116.0, 115.1, 69.4, 66.3, 63.5, 59.3, 48.8, 48.4, 46.1, 45.9, 39.7, 39.3, 35.4 (2C), 31.9, 31.8, 29.3, 29.2, 27.7, 26.9, 26.7, 26.6, 25.8, 25.7, 22.1 (2C), 17.8, 17.7 ppm. IR (film): ν = 2978 (w), 2918 (w), 1730 (s), 1665 (s), 1545 (s), 1432 (s), 1374 (w), 1374 (m), 1223 (w), 1132 (w), 1001 (w), 919 (m), 633 (w), 423 (w) cm^−1^.

MS (ESI, 70 eV): *m*/*z* (%) = 482 (1), 429 (16), 407 (100) [M + Na]^+^, 385 (58), [M + H]^+^, 327 (1), 261 (16), 249 (6), 219 (1). HRMS (ESI, C_24_H_32_O_4_) calculated ([M + Na]^+^): 407.2193; found: 407.2206.

PPAP23 Rf = 0.24 (*iso*-hexane/ethyl acetate 19:1). ^1^H-NMR (600 MHz, D_2_O) δ = 18.65 (s, 0.6H), 18.04 (s, 0.4H, b), 5.19–5.09 (m, 1H), 4.87–4.78 (m, 1H), 4.74–4.65 (m, 0.4H), 4.68–4.59 (m, 0.6H), 2.68–2.38 (m, 4H), 2.60 (s, 1.8H), 2.50 (s, 1.2H), 2.19–1.98 (m, 3H), 1.97–1.71 (m, 1H), 1.69–1.62 (m, 12H), 1.54 (s, 3H), 1.46 (s, 1.8H), 1.43 (s, 1.2H), 1.42–1.31 (m, 1H), 1.16 (s, 3H), 1.00 (s, 1.8H), 0.95 (s, 1.2H) ppm. ^13^C-NMR (151 MHz, D_2_O) δ = 208.1, 207.6, 200.9, 200.3, 199.4, 199.3, 195.4, 194.0, 134.6, 134.5, 134.2 (2C), 133.0, 132.9, 123.7, 123.4, 119.8, 119.4, 119.3, 118.8, 115.7, 114.5, 68.8, 65.9, 64.2, 59.8, 48.5, 48.0, 46.1, 45.8, 39.5, 39.3, 29.5, 29.3, 29.2, 29.1, 27.8, 26.8 (2C), 26.7, 26.6, 26.5, 26.0 (4C), 25.9, 25.8, 22.0, 21.9, 18.1, 18.0, 17.9 (2C), 17.8, 17.7 ppm. IR (film): ν = 2970 (m), 2916 (m), 2979 (w), 1731 (m), 1665 (s), 1548 (s), 1439 (s), 1395 (m), 1375 (m), 1360 (w), 1223 (w), 1137 (w), 1110 (w), 1073 (w), 1028 (w), 985 (w), 961 (w), 840 (w), 634 (w), 441 (w) cm^−1^. HRMS (ESI neg, C_28_H_40_O_4_) calculated ([M–H]^−^): 439.2843; found: 439.2869.

PPAP53 ^1^H-NMR (600 MHz, D_2_O) δ = 5.64 (ddt, J = 16.8, 10.4, 6.4 Hz, 1H), 5.41 (ddt, J = 16.8, 10.4, 6.3 Hz, 1H), 5.07–4.81 (m, 5H), 3.65 (ddd, J = 6.7, 4.2, 2.5 Hz, 1H), 2.49 (t, J = 10.6 Hz, 2H), 2.35 (d, J = 6.5 Hz, 2H), 2.15 (s, 3H), 2.01–1.91 (m, 2H), 1.83–1.75 (m, 1H), 1.57 (s, 3H), 1.42 (s, 3H), 1.08 (s, 2H), 0.82 (s, 2H) ppm. ^13^C-NMR (151 MHz, D_2_O) δ = 216.4, 203.6, 193.0, 192.9, 135.3, 135.0, 133.7, 124.9, 122.1, 117.1, 116.7, 67.7, 60.7, 47.6, 45.7, 39.2, 36.0, 30.6, 30.5, 28.8, 26.1, 24.9, 22.2, 17.2 ppm. IR (film): ν = 2973 (w), 2924 (w), 2873 (w), 2055 (w), 2030 (w), 2009 (w), 1711 (w), 1641 (s), 1579 (s), 1533 (s), 1473 (w), 1454 (m), 1368 (s), 1341 (s), 1198 (m), 1136 (w), 1064 (w), 996 (w), 912 (s), 881 (w) cm^−1^. MS (ESI neg, 70 eV): *m*/*z* (%) = 1190 (4), 835 (4), 789 (49), 709 (5), 451 (10), 383 (74) [M–Na]^-^, 341 (6), 280 (100). HRMS (ESI neg, C_24_H_31_O_4_Na) calculated ([M–Na]^−^): 383.2228; found: 383.2234.

^1^H- and ^13^C-NMR spectra of PPAP53 is shown in Figure S1, and HPLC purity of PPAP 22 and 53 is shown in Figure S2.

### 4.3. Bacterial Growth Kinetics

*S. aureus* cells grown overnight in TSB were adjusted to OD = 0.01 in a 48-well plate, and 1X MIC PPAP53 and/or 1% BSA were added to the culture. The bacterial growth was measured using Varioskan LUX Multimode Microplate Reader. The instrument carries out a kinetic measurement at optical density 578 nm, every 1 h for a total of 24 h, at 37 °C with continuous shaking.

### 4.4. Antibiotic Susceptibility Testing

The susceptibility of bacteria towards an antimicrobial compound was measured through the minimal inhibitory concentration (MIC) test [47]. The MIC values were determined by the method of microdilution, following the guidelines of the Clinical and Laboratory Standards Institute outlined in document M07-A9 [48]. Here, PPAP23/53 were serially diluted in MHB to a volume of 50 μL in a 96-well microtiter plate, to which 50 μL of 10^6^ CFU/mL USA300 were added. USA300 without PPAP served as the positive control, and MHB alone served as the negative control. The plate was then incubated for 18 h at 37 °C with continuous shaking. The lowest concentration of either PPAP that inhibited the visible growth of a microorganism was considered the MIC [49].

### 4.5. Galleria Mellonella Infection Model

The larvae of *Galleria mellonella* purchased from Reptilienkosmos (Waldkirch, Germany) were grouped based on their weights. *S. aureus* USA300 cells were grown overnight in TSB, washed with PBS and adjusted to an OD_578_ of 0.1. Ten μL of bacterial suspension corresponding to 10^6^ CFU of USA300 was injected into the last right proleg of each larva, and 10 μL of either PPAP23 or PPAP53 corresponding to a dose of 20 mg/kg was injected into the left proleg of each larva in the treatment group. Untreated larvae and larvae injected with PBS served as control groups. Ten larvae in each group were monitored over 5 days at 37 °C. The experiment was carried out three times, and the survival curves of the larvae were plotted by GraphPad Prism [50].

### 4.6. Ex Vivo Killing Assay of Galleria Mellonella Larvae

The *Galleria mellonella* larvae were crushed, and coelomic fluid was extracted and sterile filtered. A single larva yielded approximately 100 μL of liquid. To mimic the in vivo larva infection studies, the same quantity of PPAP and same CFUs of bacterial suspension were used for the ex vivo killing assay. Here, 10^6^ CFUs of USA300 and 10 μg of PPAP were co-incubated with 100 μL of coelomic fluid in a 48-well plate. Untreated coelomic fluid and coelomic fluid treated with phosphate-buffered saline (PBS) served as controls. The plate was incubated overnight at 37 °C, with a shaking speed of 20 rpm resembling the movement of larvae. The bacteria in each group were quantified by the drop plate method [51].

### 4.7. In Silico Docking of PPAP with Human Serum Albumin (HSA)

Preparation of ligand and protein structure: A well-defined crystal structure of HSA was downloaded from a protein data bank (PDB ID: 1E78) [52]. To obtain a clean structure of the HSA protein, we removed from the crystal structure the calcium and acetate ions, along with the water molecules used for crystallization. The 2D structure of PPAP was created, and its geometry optimized through energy minimization using Chem3D 16.0. The structures were visualized and verified with PyMOL software. Using AutoDock Tools, PDBQT files for the protein and ligands were prepared, and blind docking was performed with AutoDock Vina. During PDBQT file preparation, polar hydrogens and Kollman charges were added to both the protein and ligand. In the docking process, serum albumin was treated as a rigid structure, while PPAP was treated as flexible [53].

### 4.8. Mouse Model for Hematogenous S. aureus Arthritis

Female NMRI mice (6–7 weeks) were used for the experiment. Mice were kept under standard environmental conditions of temperature and light and were fed laboratory chow and water ad libitum. Pre-made batches of bacteria (Newman strain) were thawed, washed and diluted to the desired concentration. Ten NMRI mice (5 mice/group) were inoculated intravenously (i.v.) into the tail vein with 0.2 mL of *S. aureus* Newman with the expected dose (4 × 10^6^ cfu/mouse). PPAP23 was dissolved in 0.5% Tween 80 in sterile PBS. The mice were injected with 0.2 mL of the PPAP23 (100 µg/mouse) or the same volume of vehicle intraperitoneally every 12 h, starting at day 2 and continuing until day 7 after infection. The mice were regularly weighed and examined for clinical arthritis by observers blinded to the groups. Observers blinded to the treatment groups visually inspected all 4 limbs of each mouse [54]. The arthritis index was constructed by adding the scores from all 4 limbs for each animal as described before. On day 10, the mice were sacrificed, and kidneys were aseptically removed and blindly assessed by one investigator (T.J.) for abscesses. A scoring system ranging from 0 to 3 was used (0—healthy kidneys; 1—1 to 2 small abscesses on kidneys without structure changes; 2—more than 2 abscesses, but <75% kidney tissue involved; and 3—large amounts of abscesses with >75% kidney tissue involved) [54]. Thereafter, the kidneys were homogenized, and then, up to 5 serial dilutions 1:10 in PBS were performed, followed by spreading of 100 µL of bacterial suspension onto horse blood agar plates. The plates were cultured for 24 h at 37 °C and the bacteria quantified as CFUs.

### 4.9. Mouse Model of Subcutaneous Abscess Formation

Six–eight-week-old, female C57B1/6J mice were obtained from Envigo. A total of 10^5^ CFUs of *S. aureus* USA300 were mixed with sterile dextran beads (Cytodex 1, Sigma, Germany), and the mixture (0.2 mL) was injected subcutaneously (s.c.) into the shaved flanks of mice as previously described [18]. Two hours and 24 h after infection, mice were treated either with PBS or with PPAP53 (500 µg/kg) in PBS using filter paper disks, Fixomull stretch and Finn chambers under anesthesia (isofluorane). Mice were euthanized 48 h after infection. The abscesses were isolated, homogenized, plated on agar plates, and cfu was determined. Since a MOI of 10^5^ was applied, defined abscesses could be excised, and no dermonecrosis was visible after 48 h, which fits to previously described experiments [18].

### 4.10. IC25 Values of PPAP23 against Some of the Anaerobic Bacterial Strains

PPAP23 was diluted in dimethyl sulfoxide (DMSO) to a concentration of 4.5, 2.2, 0.4 and 0.2 mM (≤100×) in a v-bottom 96-well plate (Greiner Bio-One, Frickenhausen, Germany) and stored at −20 °C. Modified Gifu Anaerobic Medium broth (mGAM) (produced by Nissui Pharmaceuticals, Tokyo, Japan) was used for preparing drug plates (u-bottom plates, Nunclon delta surface, Thermo Fisher, Roskilde, Denmark), due to the robust growth of our selected species of the gut microbiome and enteropathogens. The drug plates contained 50 µL/well of 2× concentrated PPAP23, were sealed with Aluminum Foil Lids and stored at −20 °C max. 4 weeks until use. Consumables and medium were pre-reduced at least 2 days before inoculation in an anaerobic chamber (Coy Laboratory Products Inc., Grass Lake, MI, USA, 2% H_2_, 12% CO_2_, rest N_2_). Bacteria were grown twice overnight in mGAM under anaerobic conditions, and drug plates were thawed and brought in the anaerobic chamber overnight. The second overnight culture was diluted in mGAM to an OD of 0.02, and 50 µL was added to all wells of the drug plate to reach a total volume of 100 µL/well containing 1%DMSO, bacteria with an OD of 0.01 and 45, 22, 4 and 2 µM PPAP23, respectively. In the case of Bilophila wadsworthia, mGAM was supplemented with 60 mM sodium formate and 10 mM taurine. Plates were sealed with breathable membranes and incubated at 37 °C. We measured the OD at 578 nm after a 60 s shaking step for 20 h by using a microplate spectrophotometer (Epoch2, BioTek, Gen5 software, version 3.05) and an automated microplate stacker (BioStack 4, BioTek, München, Germany). Three biological replicates were tested, and growth curves were analyzed according to [55] with a cutoff of 25% (drug concentration inhibiting 25% of bacterial growth).

### 4.11. Quantification of Cytokines Using Enzyme-Linked Immunosorbent Assay

The levels of Interleukin 6 [IL6] and keratinocyte-derived chemokine (KC) from blood plasma of NMRI mice infected intravenously with *S. aureus* Newman were analyzed using a DuoSet ELISA kit [R&D Systems, Abingdon, UK] as per the manufacturer’s instructions.

## 5. Conclusions

We show here that a systemic application of PPAP23 and 53 is restricted due to their neutralization by serum albumin, but we also show that that they have no recognizable negative effects in the animal models, which is a prerequisite for potential application. There are numerous examples of drugs that are neutralized by albumin and successfully in use for the treatment of certain skin diseases or for decolonization. For example, a topical ointment of mupirocin is successfully used to decolonize *S. aureus* from nasal carriers. Although mupirocin binds to albumin to a high degree (95%), and its activity is reduced 10 to 20-fold by human serum, it is a very useful antibiotic [56]. Mupirocin, ozenoxacin (a topical quinolon), retapamulin or fusidic acid are also used to treat impetigo [57,58,59,60]. Applications in this direction can also be imagined for PPAP23 and 53, especially, when one considers that mupirocin-resistant *S. aureus* strains are already spreading. From over 100 derivatives of PPAPs, we have singled out two with particularly good antimicrobial and well-tolerated properties with great potential for topical application.

## Figures and Tables

**Table 1:** Impact of serum components on PPAP53 activity against *S. aureus* USA300.

Medium	MIC (µg/mL)		
	PPAP23	PPAP53	Vancomycin
MHB	1	0.5	1
MHB + 25% FBS	32	32	2
MHB + 0.5% BSA	4	8	1
MHB + 1% BSA	8	8	1
MHB + 2.5% BSA	16	16	1
MHB + 5% BSA	32	32	2
MHB + 1% IgG	1	0.5	1
MHB + 2.5% IgG	1	0.5	1
MHB + 1% Fg	1	0.5	1
MHB + 2.5% Fg	1	0.5	1

**Table 2:** IC_25_ values of PPAP23 and PPAP53 against some of the anaerobic bacterial strains.

Strains		PPAP23 IC_25_ ^a^ (µM)	PPAP53 IC_25_ (µM)
Pathogenic G (+) gut anaerobes	*Clostridium difficile*	<2	>80
	*Clostridium perfringens*	<2	5
	*Ruminococcus gnavus*	<2	5
	*Clostridium ramosum*	<2	10
Commensal gut anaerobes (+/−)	*Streptococcus salivarius*	<2	5
	*Dorea formicigenerans*	4	10
	*Streptococcus parasanguinis*	4	10
	*Roseburia intestinalis*	4	10
	*Coprococcus comes*	4	10
	*Collinsella aerofaciens*	4	10
	*Eubacterium rectale*	4	5
	*Clostridium bolteae*	22	10
	*Parabacteroides merdae*	22	5
	*Clostridium saccharolyticum*	22	10
	*Fusobacterium nucleatum* subsp. Nucleatum	>45	40
	*Bacteroides vulgatus*	22	5
	*Bacteroides uniformis*	>45	10
	*Bacteroides thetaiotaomicron*	>45	20
	*Bacteroides fragilis* NT	22	10
Pathogenic G (–) gut anaerobes	Yersinia pseudotuberculosis	>22	2.5
	Yersinia enterocolitica WA-314	>45	>80
	Vibrio cholerae	>45	80
	Shigella sonnei 53G	>45	>80
	Shigella flexneri	>45	>80
	Salmonella enterica Typhimurium LT2	>45	>80
	Salmonella enterica Typhimurium	>45	>80

^a^—We use IC25 because a growth inhibition of 25% is already enough to create an unbalance in a community. Bacteria do not need to die; they only need to be inhibited in their normal growth behavior resulting in being unable to support others or to close niches which should be closed for pathogenic bacteria.

## Data Availability

The data supporting the findings of this study have been previously published in the doctoral thesis of the first author.

## Supplementary Materials

The following supporting information can be downloaded at: https://www.mdpi.com/article/10.3390/ijms25158023/s1.
