# Supplementary material for: Antimicrobial Evaluation of Two Polycyclic Polyprenylated Acylphloroglucinol Compounds: PPAP23 and PPAP53
Source: Int J Mol Sci. 2024 Jul 23;25(15):8023. doi: 10.3390/ijms25158023 (PMC11312133; doi:10.3390/ijms25158023)
Supplement: Supplementary file 1 [file ijms-25-08023-s001.zip › ijms-3082759-supplementary.pdf]

Supplementary Data

Table S1. Tested ligands (FDA approved drugs) binding to HSA and their binding pockets [17].

No.	Drugs tested	Binding pocket of HSA
1	Diffusinal	IIIA, IIA-IIB
2	Iodipamide	IIA, Cleft
3	Azapropazone	IIA, IB
4	Ibuprofen	IIIA, IIA-IIB
5	Diazepam	IIIA
6	Indomethacin	IIA, IB
7	Oxyphenbutazone	IIA, IIIB
8	Phenylbutazone	IIA
9	Warfarin	IIA
10	3,5 diidosalicylic acid	IIA
11	Hemin	IB

Table S2. Colony-forming units (CFUs) isolated from USA300-induced abscesses after 48 h treatment with +/- PPAP53 (500 mg/kg body weight). Left side: CFUs of four control abscesses. Right side: CFUs mean values. Mouse 1-9 = M1-M9.

	**USA300**	**+PPAP53**		**USA300**	**+PPAP53**
		500µg/kg			500µg/kg
M 1	270000000	137000000	**M 1**	232500000	126500000
	230000000	139000000			
	230000000	120000000			
	200000000	110000000			
M 2	300000000	167000000	**M 2**	339500000	173250000
	330000000	200000000			
	385000000	176000000			
	343000000	150000000			
M3	224000000	230000000	**M3**	274250000	208750000
	253000000	210000000			
	290000000	175000000			
	330000000	220000000			
M 4	225000000	235000000	**M 4**	195250000	241750000
	193000000	255000000			
	173000000	268000000			
	190000000	209000000			
M 5	453000000	113000000	**M 5**	438000000	112625000
	423000000	128000000			
		96500000			
		113000000			

M6	392000000	325000000	**M6**	363750000	341500000
	422000000	269000000			
	298000000	417000000			
	343000000	355000000			
M7	314000000	219000000	**M7**	332000000	200250000
	324000000	219000000			
	385000000	195000000			
	305000000	168000000			
M8	410000000	296000000	**M8**	389000000	279250000
	396000000	295000000			
	360000000	332000000			
	390000000	194000000			
M 9	282000000	191000000	**M 9**	328250000	258750000
	300000000	255000000			
	373000000	322000000			
	358000000	267000000			

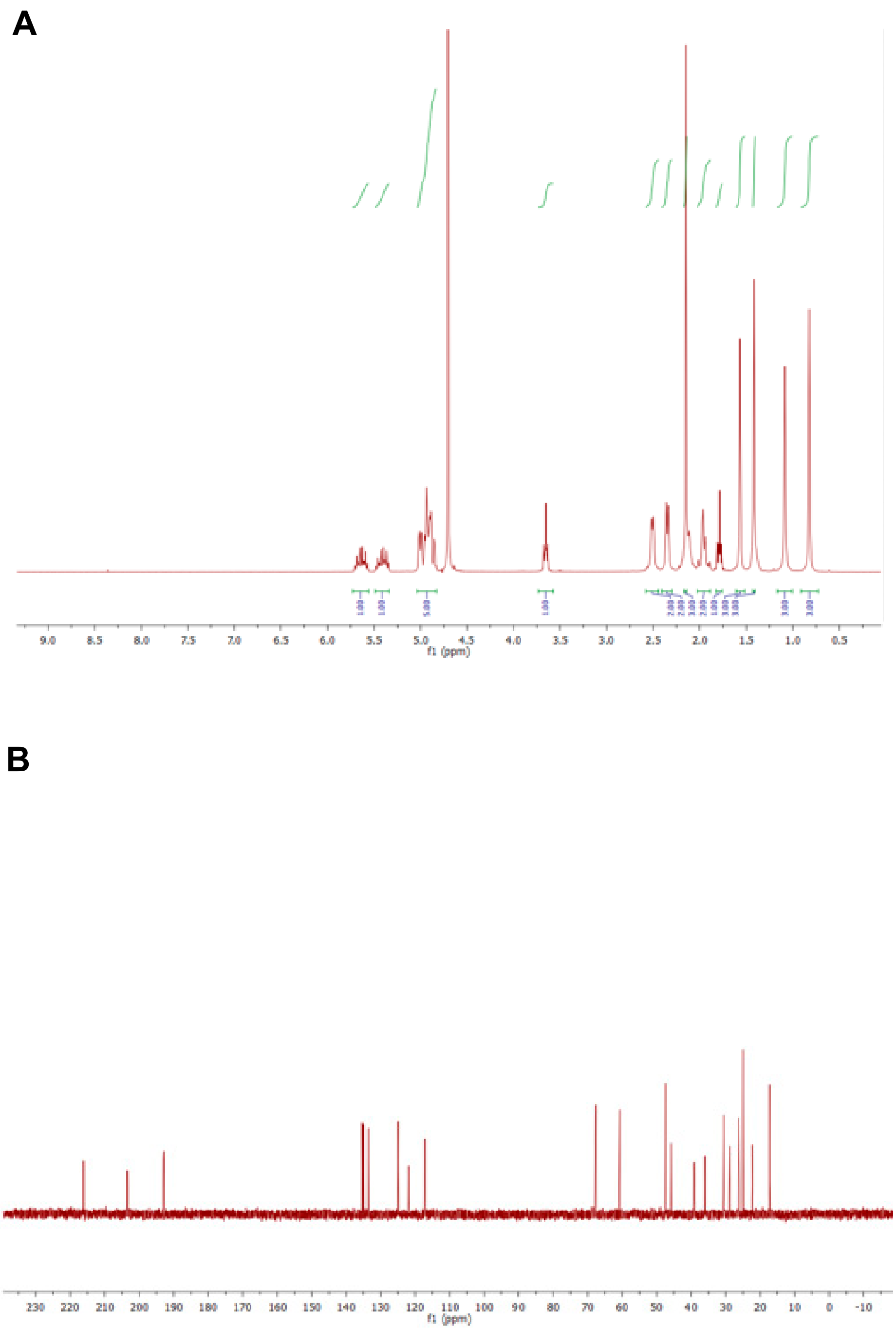

Figure S1. (A) ^{1}H- and (B) ^{13}C-NMR spectra of PPAP53.

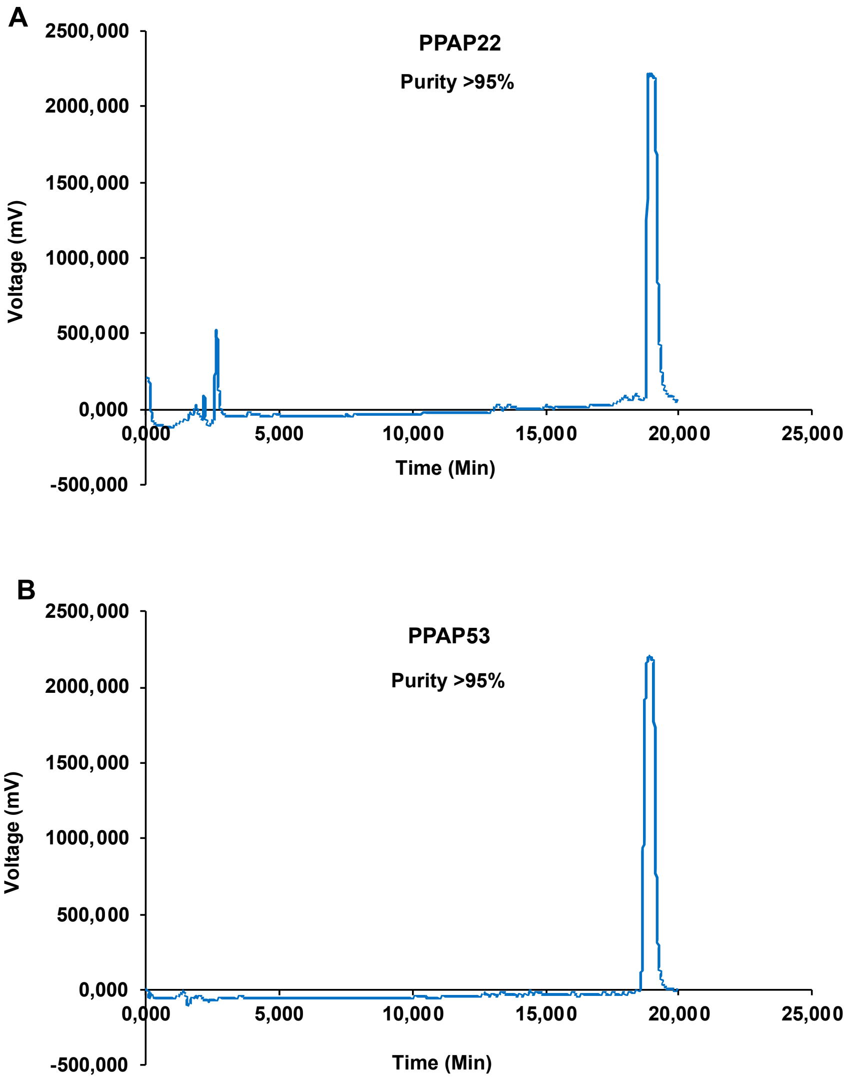

Figure S2. HPLC analysis of (A) PPAP22 and (B) PPAP53. All compounds are >95% pure by HPLC analysis.

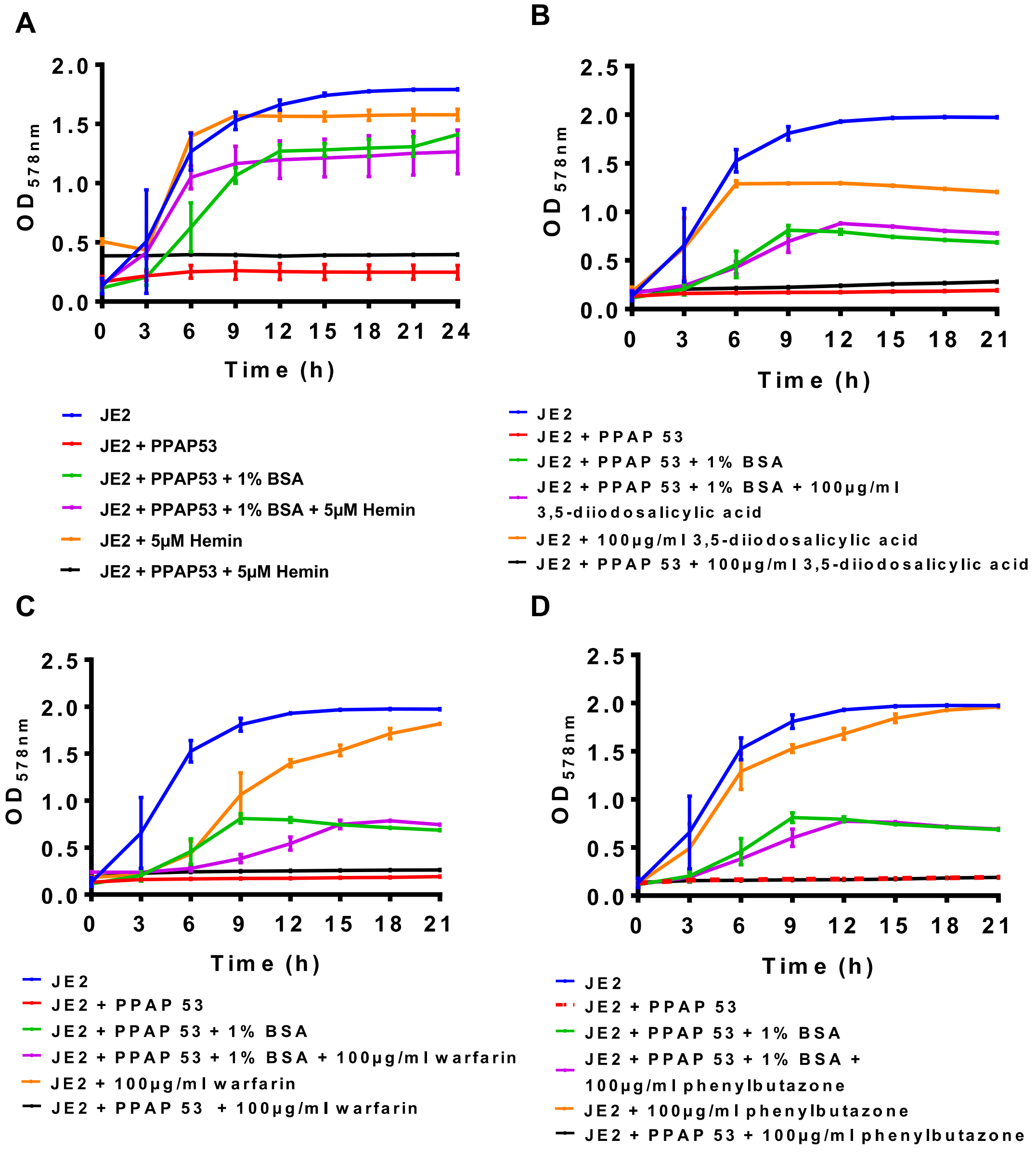

Figure S3. Adding known BSA ligands did not enhance the *in vitro* bactericidal activity of PPAP53 in the presence of BSA. *S. aureus*, cultured overnight in TSB, was inoculated to an OD of 0.01 in a 48-well plate. 1X MIC of PPAP53 and/or 1% BSA with or without ligands were added to observe the effect on bacterial growth using a Varioskan LUX Multimode Microplate Reader. This instrument recorded the optical density at 578 nm every hour for 24 hours at 37°C with continuous shaking. The ligands tested were (A) Hemin, (B) 3,5-diiodosalicylic acid, (C) Warfarin, and (D) Phenylbutazone.

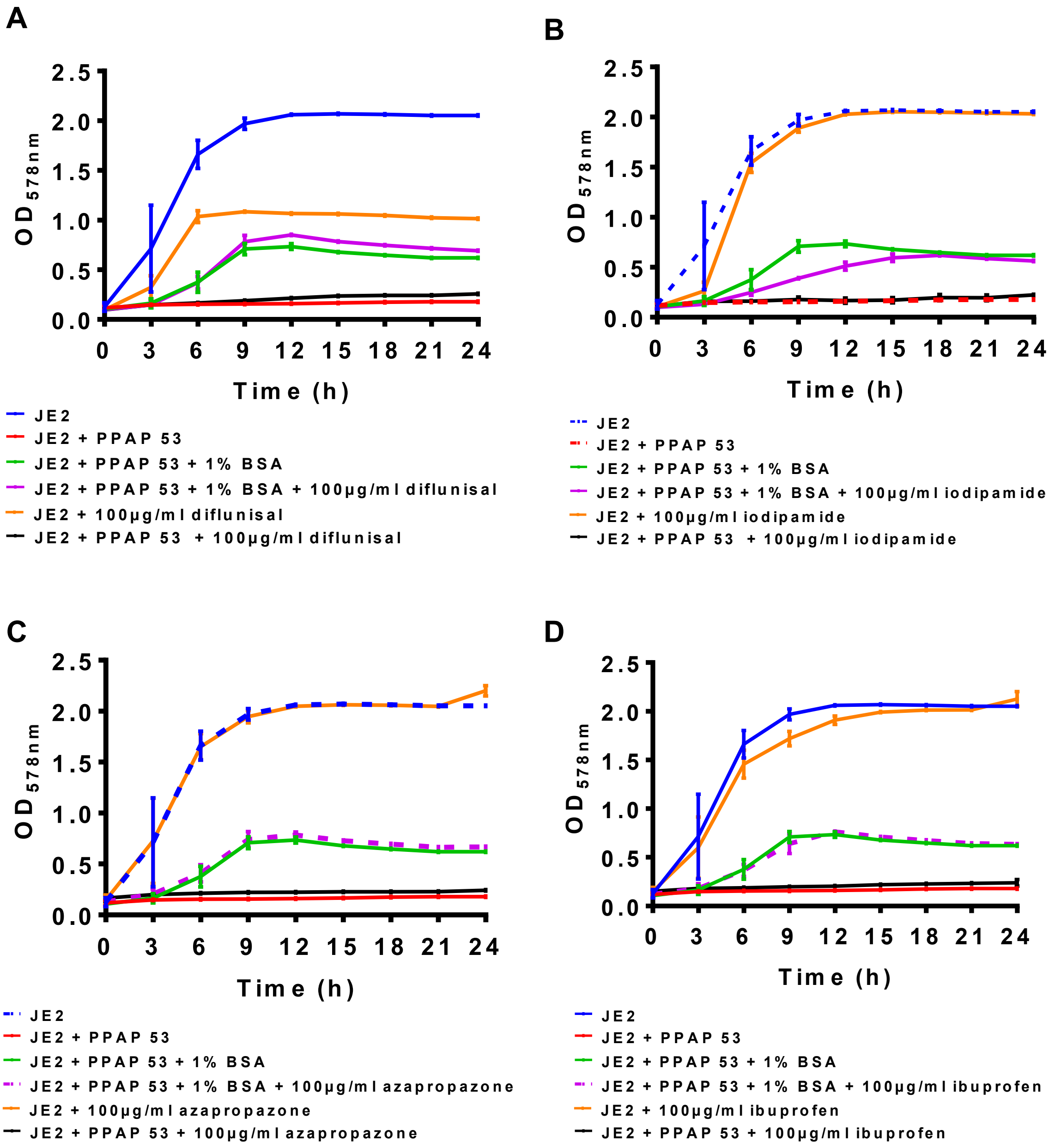

Figure S4. Adding known BSA ligands did not enhance the in vitro bactericidal activity of PPAP53 in the presence of BSA. *S. aureus*, cultured overnight in TSB, was inoculated to an OD of 0.01 in a 48-well plate. 1X MIC of PPAP53 and/or 1% BSA with or without ligands were added to observe the effect on bacterial growth using a Varioskan LUX Multimode Microplate Reader. This instrument recorded the optical density at 578 nm every hour for 24 hours at 37°C with continuous shaking. The ligands tested were (A) diffusinal, (B) iodipamide (C) azapropazone and (D) ibuprofen.

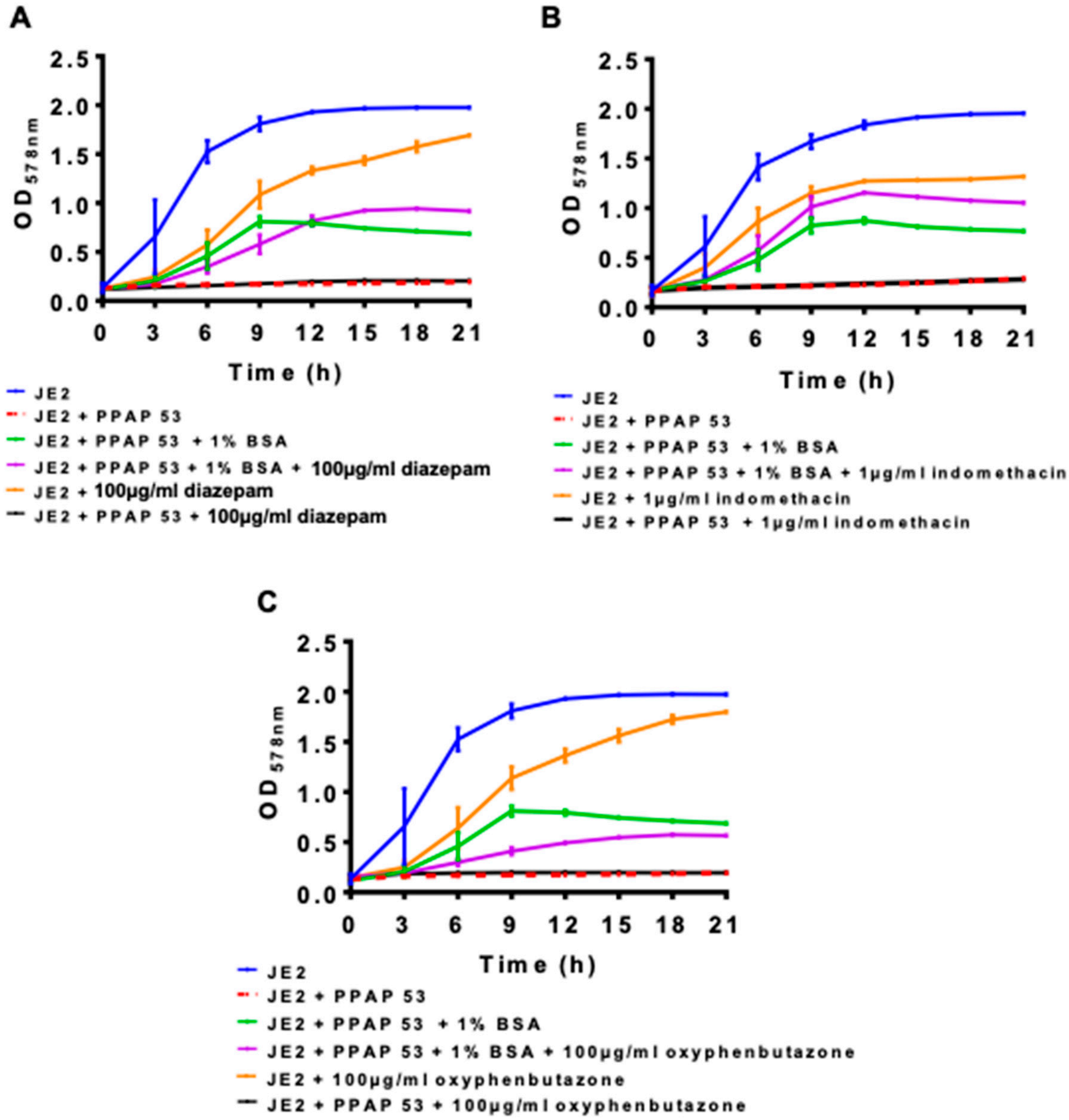

Figure S5. Adding known BSA ligands did not enhance the in vitro bactericidal activity of PPAP53 in the presence of BSA. *S. aureus*, cultured overnight in TSB, was inoculated to an OD of 0.01 in a 48-well plate. 1X MIC of PPAP53 and/or 1% BSA with or without ligands were added to observe the effect on bacterial growth using a Varioskan LUX Multimode Microplate Reader. This instrument recorded the optical density at 578 nm every hour for 24 hours at 37°C with continuous shaking. The ligands tested were (A) diazepam (B) indomethacin (C) oxyphenbutazone.
